# Conventional Hospitalization versus Sequential Outpatient Parenteral Antibiotic Therapy for *Staphylococcus aureus* Bacteremia: Post-Hoc Analysis of a Multicenter Observational Cohort

**DOI:** 10.3390/antibiotics12010129

**Published:** 2023-01-09

**Authors:** Nerea Castillo-Fernández, Pedro María Martínez Pérez-Crespo, Elena Salamanca-Rivera, Laura Herrera-Hidalgo, Arístides de Alarcón, María Dolores Navarro-Amuedo, Teresa Marrodán Ciordia, María Teresa Pérez-Rodríguez, Juan Sevilla-Blanco, Alfredo Jover-Saenz, Jonathan Fernández-Suárez, Carlos Armiñanzas-Castillo, José María Reguera-Iglesias, Clara Natera Kindelán, Lucía Boix-Palop, Eva León Jiménez, Fátima Galán-Sánchez, Alfonso del Arco Jiménez, Alberto Bahamonde-Carrasco, David Vinuesa García, Alejandro Smithson Amat, Jordi Cuquet Pedragosa, Isabel María Reche Molina, Inés Pérez Camacho, Esperanza Merino de Lucas, Belén Gutiérrez-Gutiérrez, Jesús Rodríguez Baño, Luis Eduardo López Cortés

**Affiliations:** 1 Unidad de Medicina Tropical, Hospital de Poniente, 04700 Almería, Spain; 2 Unidad de Enfermedades Infecciosas y Microbiología, Hospital Universitario Nuestra Señora de Valme, 41014 Sevilla, Spain; 3 Unidad Clínica de Enfermedades Infecciosas y Microbiología, Hospital Universitario Virgen Macarena/Departamento de Medicina, Facultad de Medicina, Universidad de Sevilla/e Instituto de Biomedicina de Sevilla (IBiS)/CSIC, 41009 Sevilla, Spain; 4 Centro de Investigación en Red de Enfermedades Infecciosas (CIBERINFEC), Instituto de Salud Carlos III, 28029 Madrid, Spain; 5 Unidad de Farmacia, Hospital Universitario Virgen del Rocío, 41013 Seville, Spain; 6 Clinical Unit of Infectious Diseases, Microbiology and Preventive Medicine, Institute of Biomedicine of Seville (IBiS), University Hospital Virgen del Rocío/CSIC/University of Seville, 4103 Seville, Spain; 7 Departamento de Microbiología Clínica, Complejo Asistencial Universitario de León (CAULE), 24071 León, Spain; 8 Unidad de Enfermedades Infecciosas, Servicio de Medicina Interna, Hospital Álvaro Cunqueiro, 36312 Vigo, Spain; 9 Unidad de Gestión Clínica de Enfermedades Infecciosas y Microbiología, Hospital Universitario Jerez de la Frontera, 11407 Cádiz, Spain; 10 Unidad Funcional de Infecciones Nosocomiales, Hospital Arnau de Vilanova, 25198 Lérida, Spain; 11 Unidad de Microbiología, Instituto de Investigación Sanitaria del Principado de Asturias (ISPA), Hospital Universitario Central de Asturias, 33011 Oviedo, Spain; 12 Unidad de Enfermedades Infecciosas, Hospital Universitario Marqués de Valdecilla, Universidad de Cantabria, IDIVAL, 39008 Santander, Spain; 13 Servicio de Enfermedades Infecciosas, Hospital Regional Universitario de Málaga/IBIMA, 29010 Málaga, Spain; 14 Unidad Clínica de Enfermedades Infecciosas, Hospital Universitario Reina Sofía, 14004 Córdoba, Spain; 15 Unidad de Enfermedades Infecciosas, Hospital Universitari Mútua de Terrassa, 08221 Barcelona, Spain; 16 Unidad de Gestión Clínica de Microbiología, Hospital Universitario Puerta del Mar, 11009 Cádiz, Spain; 17 Grupo Enfermedades Infecciosas, Servicio de Medicina Interna, Hospital Costa del Sol, 29603 Marbella, Spain; 18 Departamento de Medicina Interna, Hospital de El Bierzo, 24404 Ponferrada, Spain; 19 Unidad Gestión Clínica Enfermedades Infecciosas, Hospital Universitario Clínico San Cecilio, 18016 Granada, Spain; 20 Unidad de Medicina Interna, Fundació Hospital de l’Esperit Sant, 08923 Santa Coloma de Gramenet, Spain; 21 Departamento de Medicina Interna, Hospital Universitario de Granollers, 08402 Granollers, Spain; 22 Unidad de Enfermedades Infecciosas, Hospital Universitario Torrecárdenas, 04009 Almería, Spain; 23 Unidad de Enfermedades Infecciosas, Hospital Universitario General de Alicante, 03010 Alicante, Spain

**Keywords:** outpatient parenteral antimicrobial therapy, *Staphylococcus aureus*, bacteremia

## Abstract

It is not known whether sequential outpatient parenteral antimicrobial (OPAT) is as safe and effective as conventional hospitalization in patients with *S. aureus* bacteremia (SAB). A post-hoc analysis of the comparative effectiveness of conventional hospitalization versus sequential OPAT was performed in two prospective Spanish cohorts of patients with S. *aureus bacteremia*. The PROBAC cohort is a national, multicenter, prospective observational cohort of patients diagnosed in 22 Spanish hospitals between October 2016 and March 2017. The DOMUS OPAT cohort is a prospective observational cohort including patients from two university hospitals in Seville, Spain from 2012 to 2021. Multivariate regression was performed, including a propensity score (PS) for receiving OPAT, stratified analysis according to PS quartiles, and matched pair analyses based on PS. Four hundred and thirteen patients were included in the analysis: 150 in sequential OPAT and 263 in the full hospitalization therapy group. In multivariate analysis, including PS and center effect as covariates, 60-day treatment failure was lower in the OPAT group than in the full hospitalization group (*p* < 0.001; OR 0.275, 95%CI 0.129–0.584). In the PS-based matched analyses, sequential treatment under OPAT was not associated with higher 60-day treatment failure (*p* = 0.253; adjusted OR 0.660; % CI 0.324–1.345). OPAT is a safe and effective alternative to conventional in-patient therapy for completion of treatment in well-selected patients with SAB, mainly those associated with a low-risk source and without end-stage kidney disease.

## 1. Introduction

Outpatient parenteral antimicrobial therapy (OPAT) has been shown to be a safe choice for the treatment of a wide range of infectious diseases whenever oral therapy is not appropriate [1,2,3]. In well-selected patients, such as those who are clinically stable, and have uncomplicated bacteremia or a low-risk focus of infection, this strategy can reduce the duration of hospitalization and thus the risk of nosocomial infections, which improves quality of life and reduces costs [4,5]. The efficacy of OPAT has recently been demonstrated in infective endocarditis, even when applied with broader criteria than the standard guideline recommendations [6,7,8,9]. 

*Staphylococcus aureus* is a main cause of bacteremia in healthcare, nosocomial, and community settings, leading to a large number of secondary complications, as well as high morbidity and mortality [10,11]. There is growing interest in defining the patient profile and clinical circumstances in which sequential oral treatment or long-acting antibiotics should be used [12,13,14,15,16,17]. However, the best clinical profile for these strategies in *S. aureus* bacteremia (SAB) is not yet well established [12,13,14,15,16,17]. Thus, for a variety of reasons, many patients need to complete the entire therapeutic regimen intravenously. The need for prolonged hospitalization to receive intravenous antimicrobial therapy makes OPAT an attractive option for continuation of treatment in patients with SAB. Despite the proven safety of OPAT in other infectious diseases, few studies have shown whether it is possible to treat SAB patients in OPAT after an initial period in hospital [18,19,20,21,22,23,24,25]. The lack of evidence could be partly explained by the fact that clinical management of SAB is challenging due to its high virulence, the need for close clinical follow-up to prevent or detect early complications such as endocarditis, distant metastatic foci or relapse, or by the adverse events of first-line antibiotics [19,20,22,23,26,27]. Nevertheless, interest in the use of OPAT programs to reduce costs is growing in light of new evidence on the effectiveness and safety of intravenous therapies with a good pharmacokinetic profile, such as cefazoline or ceftriaxone [28,29,30,31]. By defining the characteristics of patients who can properly be managed with oral therapy or in an OPAT program, the management of SAB could be modified in the coming years as a way of improving the patient’s quality of life. 

The aim of this study was to compare the clinical effectiveness of conventional hospitalization versus sequential OPAT in the treatment of SAB. 

## 2. Methods

### 2.1. Study Design and Patients

This study is a post-hoc analysis of two prospective Spanish cohorts of patients with SAB. Patients who received intravenous therapy entirely in hospital (full hospitalization therapy group) were compared with those who started antibiotic treatment in hospital and continued sequential therapy in an OPAT program. Patients with SAB from the PROBAC cohort (a national, multicenter prospective observational cohort study of patients with bloodstream infections (BSI) diagnosed in 22 Spanish hospitals between October 2016 and March 2017), were eligible, and were included either in the full hospitalization or OPAT groups, as appropriate. Due to the limited number of patients in the OPAT group, patients with SAB treated in the DOMUS OPAT program, a service provided by the Virgen del Rocio and Virgen Macarena University Hospitals (both in Seville, Spain) from 2012 to 2021, were also considered eligible. The specific features of the DOMUS OPAT program have been extensively described in previous publications [28,32,33]. Patients undergoing hemodialysis after a period of conventional hospitalization were included in the full hospitalization therapy group as part of our objective to explore the effectiveness of OPAT versus standard practice.

Eligible patients were included in the analysis if they fulfilled all of the following criteria: (1) older than 14 years; (2) had monomicrobial SAB; and (3) survived at least 7 days after diagnosis of SAB. Patients who switched to oral therapy were excluded. A thirty-day follow-up of patients was carried out by the local bacteremia services. Following discharge from hospital, data were obtained by reviewing clinical records. 

### 2.2. Ethical Statement

The activity of the DOMUS OPAT program has been approved by the research ethics committees of the University Hospital Virgen del Rocio and the University Hospital Virgen Macarena. The PROBACT project was approved by the ethics committees of the participating centers, which waived the need for informed consent due to the observational design. This project has been registered in ClinicalTrials.gov (NCT03148769).

### 2.3. Variables and Definitions

Data collected (with definitions below) included: age, gender, type of hospital service, type and severity of chronic underlying diseases according to the Charlson comorbidity index [34], renal replacement therapy, type of acquisition, suspected source of infection, methicillin susceptibility, presence of complicated bacteremia, length of hospital admission, total duration of therapy, and duration of hospital treatment before inclusion to the OPAT program. The primary endpoint was treatment failure, defined as a composite of 60-day crude mortality and/or 60-day relapse. Relapse was defined as the development of a new *S. aureus* bacteremia caused presumptively by the same strain based on the antibiogram once antibiotic treatment of the initial episode had been completed. Charlson comorbidity index, both crude and age-adjusted, was calculated. Source of infection was classified as vascular catheter-related (both peripheral and central), osteoarticular, respiratory tract, intra-abdominal, skin and soft tissue, infective endocarditis, intravascular device-associated, unknown, and other (including nervous central system and urinary tract). Vascular catheter-related SAB was defined according to ECDC criteria [35]. Complicated bacteremia was defined as the presence of infective endocarditis, distant metastatic foci, persistence of positive blood cultures or fever after three or more days of adequate treatment, or the presence of any prosthesis or device (vascular, valve, orthopedic or intracardiac). Patients who received more than 10 mg of prednisone (or equivalent) for more than 3 weeks and those who had received antineoplastic chemotherapy treatment in the last month were considered immunosuppressed.

### 2.4. Statistical Analysis

Continuous variables were expressed as median and interquartile range (IQR) and categorical variables as absolute numbers and percentages. Univariate comparisons were performed by the chi-square or Fisher tests for categorical variables, and the Mann–Whitney *U* test for continuous variables. Potentially clinically relevant variables and those with a univariate *p* value < 0.2 were included in the multivariate logistic regression model to estimate the adjusted OR for treatment failure. Potential interactions were explored and included if they had a significant modifying effect. Variable selection was performed manually using a backward stepwise procedure. In addition, a propensity score (PS) for treatment in an OPAT program was calculated by developing a non-parsimonious logistic regression model, with OPAT as the dependent variable, and age, chronic heart failure, chronic renal failure, hemodialysis, chronic obstructive pulmonary disease, malignancy, vascular catheter source, methicillin-resistant *S. aureus* (MRSA), endocarditis, valve prosthesis, persistent bacteremia, persistent fever > 72 h, vascular prosthesis, orthopedic prosthesis, distant metastatic foci, and intracardiac device as predictors. The PS was used first as a covariate in multivariate analysis, then to perform a stratified analysis according to PS quartiles, and finally to match patients in OPAT or full hospitalization therapy, using the minimum absolute difference between scores and a maximum tolerance of 5%. Whenever more than one pairing was possible, selection was by simple randomization. To control for the center effect, centers were grouped by regression analysis into low- and high-risk based on 60-day mortality; this variable was included in the multivariate analysis [36]. SPSS 26.0 software and TreeNet software (Salford System) were used for statistical analysis. 

## 3. Results

**Figure 1:**
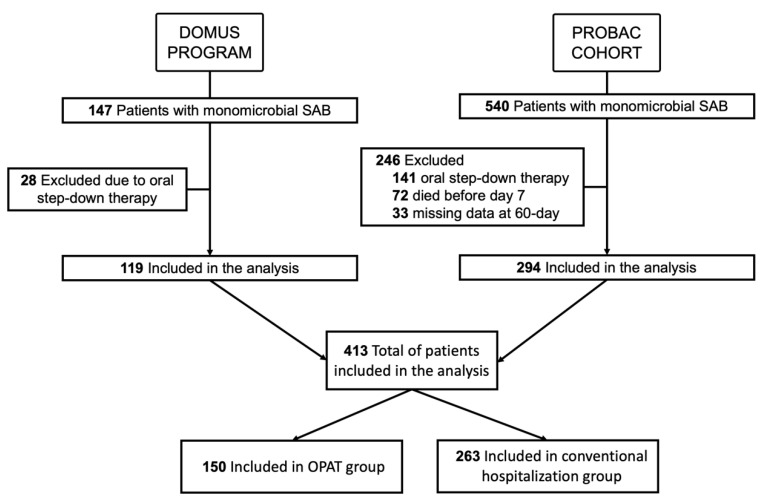
Flowchart of study.

During the study period, 147 patients from the DOMUS OPAT program and 540 from the PROBAC cohort were diagnosed with monomicrobial SAB. Patients who received oral step-down therapy (*n* = 169) or died within the first seven days of the diagnosis (*n* = 72) were excluded. Thirty-three patients were excluded because of missing data on the primary objective (see Figure 1). In total, 413 patients were included in the analysis:150 in the OPAT group and 263 in the full hospitalization therapy group. 

Table 1 shows the baseline demographics and clinical characteristics of patients included in the study, as well as a comparative analysis of the two groups. 

Overall, most cases were nosocomial or healthcare-associated (75.5%). Vascular catheter was the most frequent source (177 cases, 42.9%), followed by unknown sources (63 cases, 15.3%), and skin and soft tissue (62 cases, 15.0%). Eighty-three (20.1%) cases were methicillin-resistant. Complicated bacteremia was diagnosed in 157 patients (38.0%) and infective endocarditis in 24 patients (5.8%). Patients included in the OPAT group were younger, with lower rates of chronic renal failure but higher rates of cardiac and malignancy comorbidities. The most frequent source of bacteremia in OPAT patients was the vascular catheter (62.0% vs. 31.9%, *p* < 0.001), while an unknown source (8.7% vs. 19.9%, *p* = 0.005) and a respiratory tract focus (2.0% vs. 9.1%, *p* = 0.005) were significantly less common. Patients from the OPAT group presented higher rates of complicated bacteremia (45.3% vs. 33.8%, *p* = 0.027), especially those due to distant metastatic foci (10.0% vs. 4.6%, *p* = 0.032), the presence of a valve prosthesis (7.3% vs. 3.0%, *p* = 0.045) or an intracardiac device (12.7% vs. 4.2%, *p* = 0.001). Persistent fever >72 h was more frequent in the full hospitalization group. The median length of hospital treatment before OPAT was 10 days (IQR 6–14) with a significantly shorter duration of hospital stay. Overall, the most frequent targeted treatments were cloxacillin (*n* = 115, 27.8%), cefazolin (*n* = 107, 25.9%), daptomycin (*n* = 58, 14.0%) and vancomycin (*n* = 18, 4.4%). Other antibiotics used were linezolid (*n* = 17, 4.1%), levofloxacin (*n* = 7, 1.7%), clindamycin (*n* = 7, 1.7%), teicoplanin (*n* = 6, 1.5%), and other beta-lactams in 12 patients (4 amoxicillin clavulanic; 4 piperacillin tazobactam; 2 meropenem; and 2 ceftriaxone). Thirty-nine patients in the full hospitalization cohort (14.8%) received combination therapy, with cloxacillin plus daptomycin (*n* = 20, 4.8%), cloxacillin plus vancomycin (*n* = 6, 1.5%) and cefazolin plus daptomycin (*n* = 3, 0.7%) being the most common combinations. The remaining patients (*n* = 9, 2.2%) received a combination with an aminoglycoside and one (0.2%) daptomycin plus fosfomycin. Data on targeted therapy were missing in 27 patients (6.5%). 

In the OPAT cohort, the targeted treatment administered after hospitalization was a monotherapy regimen in all cases. The most frequent treatment was cefazolin (*n* = 77, 51.3%), followed by cloxacillin in 43 patients (28.7%), daptomycin (*n* = 18, 12.0%), vancomycin (*n* = 4, 2.7%), and teicoplanin (*n* = 4, 2.7%). Overall, the rate of treatment failure at 60 days was 26.2% (108/413), being lower in the OPAT group than in the full hospitalization group (*p* < 0.001). Table 2 shows the bivariate analysis of 60-day treatment failure. 

Age over 65 years, chronic obstructive pulmonary disease, medical department as service in charge, intra-abdominal or unknown source of infection, MRSA strain and complicated bacteremia were statistically associated with higher crude rates of 60-day treatment failure. However, the number of treatment failure events at 60 days was lower in vascular catheter-related bacteremia (*p* = 0.001). The multivariate logistic analysis is shown in Table 3. 

**Figure 2:**
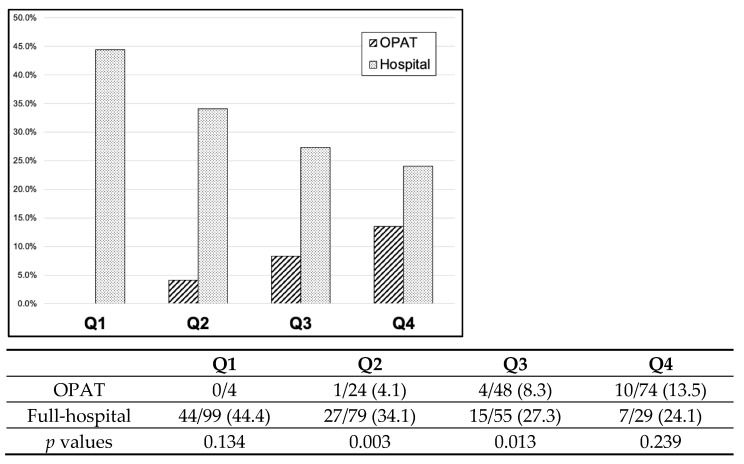
Sixty-day treatment failure stratified according to propensity score quartiles for being treated in the OPAT program (Q1, Q2, Q3, Q4). Data in the table are number of failures (death and/or relapse) / number of patients in each therapeutic modality group (percentage).

No significant interactions were found between covariates. A PS for treatment in an OPAT program was calculated; the model showed a *p* value of 0.180 for the Hosmer–Lemeshow goodness-of-fit test and an area under the receiver operating characteristic curve of 0.829 (95% CI 0.790–0.868), showing high predictive ability for the observed data. In multivariate analysis, including PS and center effect as covariates, 60-day treatment failure was lower in the OPAT group than in the full hospitalization therapy group (*p* = 0.001; OR 0.275; 95% CI 0.129–0.584). Patients were then stratified according to PS quartiles (Figure 2). 

Except for the 1st and 4th quartile, where differences did not reach statistical significance, the rates of treatment failure were lower in patients treated in OPAT. Cases from the OPAT and full hospitalization groups were then matched according to PS, which yielded 101 matched pairs (202/413, 48.9% of cases). As shown in Table 4, the baseline characteristics of the matched patients were similar and confirmed that they were well balanced. In this analysis, the sensitivity of the association between treatment in OPAT and failure was less precise, but no association with an increased risk of treatment failure at 60 days could be established (*p* = 0.253; adjusted OR 0.660; % CI 0.324–1.345).

## 4. Discussion

The results of this study suggest that follow-up treatment in an OPAT program in well-selected patients with SAB is safe and is not associated with higher rates of mortality and/or relapse compared with a full course of in-hospital intravenous treatment. Given the characteristics of patients in the OPAT group, it would be especially applicable to younger patients without end-stage kidney disease, and with SAB secondary to “low-risk” sources, such as catheter-related bloodstream infections. The strict selection of patients eligible for OPAT would clearly explain their more favorable prognosis in this analysis. However, the aim of this study was to assess whether the lower risk population can be successfully treated in OPAT. After considering possible limitations and uncontrolled confounders, and balancing all these features according to PS and center effect, no association or trend between sequential OPAT and worse outcome was found. 

A number of studies have analyzed the effectiveness and safety of OPAT in clinically stable patients with SAB [6,19,21,25,26,30]. Rehm et al. [26] conducted a post-hoc analysis of a randomized trial comparing daptomycin with standard therapy. In their study, patients with sequential treatment in OPAT were selected from the original cohort but, unlike our study, patients with clearance creatinine < 30 mL/min, osteomyelitis or pneumonia were excluded. The treatment failure rates (9.7% in the OPAT group vs. 26.8% in the inpatient group) were similar but slightly lower than those found in our study, particularly in patients who completed the entire treatment in the hospital setting. The rates of complicated bacteremia and endocarditis were similar to those in our study, reflecting the high rate of clinical success in OPAT. One difference is that, although patients were selected from a randomized trial, the trial was not designed to analyze outcomes in OPAT, so the baseline and clinical characteristics of patients between the two groups may have been unbalanced and not statistically controlled. Several retrospective studies have reported similar mortality or treatment failure rates, which were even lower when more restrictive criteria were used or deep-seated infections were excluded [19,21,25,30]. In those studies, readmissions and deaths were not directly related to OPAT complications (e.g., worsening of neoplastic disease or baseline comorbidities). Unfortunately, we were unable to collect this information in our cohort. Based on published data from previous studies, acquisition of SAB is frequently nosocomial, which worsens prognosis, increases the risk of mortality, and delays hospital discharge, as is the case in patients with catheter-related bloodstream infections [10,11]. Consequently, completion of therapy in an OPAT program to reduce hospital stay would appear to be safe. In our cohort, patients with deep-seated sources, such as respiratory tract or intra-abdominal infection; those from an unknown source; and those with end-stage kidney disease were more often fully treated in the inpatient setting, probably because outpatient management was more problematic. 

In the last decade, reported data suggest that OPAT programs are safe and effective in infective endocarditis [6,7,8]. The small number of cases in our cohort does not allow conclusions to be drawn. Recently, a prospective analysis of patients with endocarditis managed in OPAT or hospital-only programs reported that 15.4% of endocarditis cases were secondary to *S. aureus*, with no differences in efficacy or safety between the two groups [7]. In a retrospective study of patients with infectious endocarditis treated in OPAT, Htin et al. [37] reported that 41% were linked to *S. aureus*. Of 28 patients with *S. aureus* endocarditis treated in the outpatient setting, only three developed complications (readmission, relapse, or death), caused by undrained foci of infection, which may be an important consideration for admission to an OPAT program. Other studies have reported heterogeneous results, depending on clinical characteristics, which is why well-designed studies on *S. aureus* endocarditis comparing OPAT with inpatient management are needed [6,9,38]. Outpatient management in endocarditis may be safe in well-selected patients, which includes those who are clinically and hemodynamically stable, do not have end-stage liver disease, undrained abscesses or septic emboli, have no complications requiring surgery, or microorganisms that are very difficult to treat [9]. 

Our study has the intrinsic limitations of an observational study, including the fact that the selection criteria for OPAT were based on the criteria of each hospital, as well as the potential effects of unmeasured variables and residual confounders. While most of those included in the OPAT group belonged to the DOMUS OPAT program and not to the PROBAC cohort, this would not affect the conclusion that OPAT appears to be safe in well-selected patients. It is possible that some of the causes of mortality were not directly related to *S. aureus* bacteremia, but the reasons for this were not available for analysis. A strength of the study is the use of advanced statistical methods to control for confounders. Furthermore, this is, to our knowledge, the first and largest study to compare treatment for SAB in OPAT versus conventional full hospitalization without restrictive criteria, reflecting real practice.

## 5. Conclusions

In conclusion, our results suggest that OPAT is a safe and effective option for completing treatment in well-selected patients with SAB, mainly those with a low-risk source and without end-stage kidney disease, as an alternative to conventional hospital treatment. Patients with nosocomial catheter-related bacteremia may be the most suitable patients for an OPAT program in order to reduce the length of hospital stay. The need to improve the quality of life for these patients and to reduce morbidity and hospitalization costs calls for randomized trials to identify the best candidates for an OPAT program when switching to oral therapy is not an option.

## Figures and Tables

**Table 1:** Features of cases with *Staphylococcus aureus* bloodstream.

	All Cases (*n* = 413)	OPAT Group (*n* = 150)	Full Hospital-Based Therapy (*n* = 263)	*p* Value
Median age (IQR)	69 (56–79)	64 (51–77)	70 (58–79)	0.009
Male gender	283 (68.5%)	105 (70.0%)	178 (67.9%)	0.664
Comorbidities				
Charlson index ≥ 2	290 (79.2%)	110 (73.3%)	180 (68.4%)	0.296
Diabetes Mellitus	121 (29.3%)	42 (28.0%)	79 (30.0%)	0.662
COPD	57 (13.8%)	25 (16.7%)	32 (12.2%)	0.202
Chronic renal failure	102 (24.7%)	28 (18.7%)	74 (28.1%)	0.032
Chronic heart failure	91 (22.0%)	50 (33.3%)	41 (15.6%)	<0.001
Malignancy	137 (33.2%)	67 (44.7%)	70 (26.6%)	<0.001
Chronic liver disease	35 (8.5%)	12 (8.0%)	23 (8.7%)	0.794
Immunosuppressed	40 (9.7%)	16 (10.7%)	24 (9.1%)	0.611
Acquisition of infection				
Community-acquired	101 (24.5%)	30 (20.0%)	71 (27.0%)	0.112
Nosocomial/healthcare acquisition	312 (75.5%)	120 (80.0%)	192 (73.0%)	0.112
Department				
Surgery	65 (15.8%)	29 (19.3%)	36 (13.7%)	0.134
Medical	347 (84.2%)	121 (80.7%)	226 (86.3%)	0.134
Source of infection				
Vascular catheter	177 (42.9%)	93 (62.0%)	84 (31.9%)	< 0.001
Osteoarticular	24 (5.8%)	5 (3.3%)	19 (7.2%)	0.104
Unknown	63 (15.3%)	13 (8.7%)	50 (19.9%)	0.005
Respiratory tract	27 (6.5%)	3 (2.0%)	24 (9.1%)	0.005
Intra-abdominal	9 (2.2%)	1 (0.7%)	8 (3.0%)	0.165
Skin and soft tissues	62 (15.0%)	17 (11.3%)	45 (17.1%)	0.114
Endocarditis or vascular prosthesis	21 (5.1%)	9 (6.0%)	12 (4.6%)	0.523
Others	17 (4.1%)	6 (4.0%)	11 (4.2%)	0.928
Methicillin-resistant *S. aureus*	83 (20.1%)	20 (13.3%)	63 (24.0%)	0.011
Complicated bacteremia	157 (38.0%)	68 (45.3%)	89 (33.8%)	0.027
Infective endocarditis	24 (5.8%)	12 (8.0%)	12 (4.6%)	0.189
Metastatic distant foci	27 (6.5%)	15 (10.0%)	12 (4.6%)	0.032
Persistent bacteremia	45 (10.9%)	14 (9.3%)	31 (11.8%)	0.442
Persistent fever > 72h	43 (10.4%)	5 (3.3%)	38 (14.4%)	<0.001
Vascular prosthesis	12 (2.9%)	6 (4.0%)	6 (2.3%)	0.317
Valve prosthesis	19 (4.6%)	11 (7.3%)	8 (3.0%)	0.045
Orthopedic prosthesis	23 (5.6%)	12 (8.0%)	11 (4.2%)	0.104
Intracardiac device	30 (7.3%)	19 (12.7%)	11 (4.2%)	0.001
Total duration of therapy, days, median (IQR)	17 (14–27)	20 (15–29)	16 (12–22)	<0.001
Length of hospital stay, days, median (IQR)	17 (12–28)	16 (11–21)	22 (12–35)	<0.001
60-day overall mortality	93 (22.5%)	10 (6.7%)	83 (31.6%)	<0.001
Treatment failure	108 (26.2%)	15 (10.0%)	93 (35.4%)	<0.001

Data are presented as *n* (%) unless indicated otherwise. Abbreviation: OPAT—outpatient parenteral antibiotic therapy; IQR—interquartile range; COPD—chronic obstructive pulmonary disease.

**Table 2:** Bivariate analysis of variables associated with 60-day treatment failure (including 60-day mortality and/or 60-day relapse).

	Treatment Failure (*n* = 108)	Treatment Success (*n* = 305)	*p* Value	OR (95% CI)
OPAT group	15 (13.9%)	135 (44.3%)	<0.001	0.203 (0.113–0.367)
Age ≥ 65 years	79 (73.1%)	161 (52.8%)	<0.001	2.436 (1.506–3.943)
Male sex	74 (68.5%)	209 (68.8%)	0.964	0.989 (0.616–1.588)
Comorbidities				
Charlson index ≥ 2	81 (75.0%)	209 (68.5%)	0.206	1.378 (0.837–2.268)
Diabetes mellitus	29 (26.9%)	92 (30.2%)	0.516	0.850 (0.520–1.388)
COPD	21 (19.4%)	36 (11.8%)	0.048	1.804 (1.000–3.254)
Chronic renal failure	34 (31.5%)	68 (22.3%)	0.057	1.601 (0.984–2.607)
Chronic heart failure	26 (24.1%)	65 (21.3%)	0.552	1.171 (0.696–1.968)
Malignancy	39 (36.1%)	98 (32.1%)	0.450	1.194 (0.753–1.892)
Chronic liver disease	11 (10.2%)	24 (7.9%)	0.458	1.328 (0.627–2.811)
Dialysis therapy	9 (8.3%)	22 (7.2%)	0.704	1.169 (0.521–2.625)
Immunosuppression	11 (10.2%)	29 (9.5%)	0.838	1.079 (0.519–2.243)
Acquisition of infection				
Community-acquired	30 (27.8%)	71 (23.3%)	0.350	1.268 (0.771–2.085)
Department				
Medical	98 (90.7%)	249 (81.9%)	0.031	2.165 (1.061–4.417)
Source of infection				
Vascular catheter	32 (29.6%)	145 (47.5%)	0.001	0.465 (0.290–0.744)
Osteoarticular	5 (4.6%)	19 (6.2%)	0.541	0.731 (0.266–2.007)
Unknown	24 (22.2%)	39 (12.8%)	0.019	1.949 (1.108–3.427)
Respiratory tract	9 (8.3%)	18 (5.9%)	0.380	1.449 (0.631–3.331)
Intra-abdominal	6 (5.6%)	3 (1.0%)	0.012	5.922 (1.454–24.108)
Skin and soft tissues	18 (16.7%)	44 (14.4%)	0.575	1.186 (0.652–2.158)
Endocarditis or vascular prosthesis	6 (5.6%)	15 (4.9%)	0.796	1.137 (0.430–3.010)
Others	6 (4.6%)	11 (3.6%)	0.401	1.572 (0.567–4.360)
Methicillin-resistant *S. aureus*	34 (31.5%)	49 (16.1%)	0.001	2.400 (1.444–3.990)
Complicated bacteremia	55 (50.9%)	102 (33.4%)	0.001	2.065 (1.322–3.226)
Endocarditis	7 (6.5%)	17 (5.6%)	0.729	1.174 (0.473–2.914)
Metastatic distant foci	10 (9.3%)	17 (5.6%)	0.183	1.729 (0.766–3.902)
Persistent bacteremia	24 (22.2%)	21 (6.9%)	<0.001	3.864 (2.049–7.286)
Persistent fever > 72 h	20 (18.5%)	23 (7.5%)	0.001	2.787 (1.462–5.313)
Vascular prosthesis	4 (3.7%)	8 (2.6%)	0.521	1.428 (0.421–4.841)
Valve prosthesis	4 (3.7%)	15 (4.9%)	0.791	0.744 (0.241–2.291)
Orthopedic prosthesis	7 (6.5%)	16 (5.2%)	0.630	1.252 (0.501–3.131)
Intracardiac device	8 (7.4%)	22 (7.2%)	0.947	1.029 (0.444–2.385)

**Table 3:** Multivariate logistic regression analysis for OPAT program on 60-day treatment failure.

	*p* Value	Adjusted OR (95% CI) ^a^
OPAT therapy	0.001	0.275 (0.129–0.584)
Age ≥ 65 years	0.061	1.719 (0.975–3.030)
Comorbidities		
COPD	0.049	2.076 (1.002–4.299)
Chronic renal failure	0.468	1.258 (0.677–2.335)
Chronic heart failure	0.833	1.083 (0.514–2.283)
Malignancy	0.039	1.869 (1.033–3.380)
Medical department	0.036	2.455 (1.061–5.680)
Source of infection		
Vascular catheter	0.420	0.758 (0.387–1.485)
Unknown	0.506	1.265 (0.633–2.531)
Intra-abdominal	0.088	4.264 (0.807–22.525)
Methicillin-resistant *S. aureus*	0.017	2.125 (1.147–3.935)
Complicated bacteremia	<0.001	2.967 (1.722–5.109)
High-risk center	0.001	3.389 (1.629–7.049)

^a^ The propensity score for being treated in OPAT was included in the analysis.

**Table 4:** Crude and multivariate analysis of matched cases according to propensity score.

	Crude Analysis ^a^				Adjusted Analysis ^b^	
	All Cases (*n* = 202)	OPAT Group (*n* = 101)	Full Hospital-Based Therapy (*n* = 101)	*p* Value	OR Adjusted (95%CI)	*p* Value
Median age (IQR)	65 (53–76)	64 (53–77)	65 (54–76)	0.790 ^c^		
Age ≥ 65 years	102 (50.5%)	50 (49.5%)	52 (51.5%)	0.778	0.983 (0.658–1.469)	0.932
Comorbidities						
Charlson index ≥ 2	144 (71.3%)	70 (69.3%)	74 (73.3%)	0.534	0.874 (0.560–1.365)	0.554
Diabetes mellitus	61 (30.2%)	29 (28.7%)	32 (31.7%)	0.646		
COPD	24 (11.9%)	12 (11.9%)	12 (11.9%)	1.000		
Chronic renal failure	36 (17.8%)	17 (16.8%)	19 (18.8%)	0.713		
Chronic heart failure	52 (25.7%)	26 (25.7%)	26 (25.7%)	1.000		
Malignancy	82 (40.6%)	44 (43.6%)	38 (37.6%)	0.390		
Chronic liver disease	19 (9.4%)	9 (8.9%)	10 (9.9%)	0.810		
Immunosuppressed	20 (9.9%)	7 (6.9%)	13 (12.9%)	0.158		
Nosocomial/healthcare acquisition	152 (75.2%)	77 (76.2%)	75 (74.3%)	0.744		
Medical department	170 (84.6%)	84 (83.2%)	86 (86.0%)	0.578	0.882 (0.506–1.536)	0.656
Source of infection						
Vascular catheter	108 (53.5%)	56 (55.4%)	52 (51.5%)	0.573	1.109 (0.702–1.754)	0.656
Unknown	26 (12.9%)	12 (11.9%)	14 (13.9%)	0.674	1.212 (0.602–2.439)	0.590
Respiratory tract	7 (3.5%)	2 (2.0%)	5 (5.0%)	0.445 ^d^		
Intra-abdominal	3 (1.5%)	0	3 (3.0%)	0.246 ^d^		
Others ^e^	58 (28.7%)	31 (30.7%)	27 (26.7%)	0.534		
Methicillin-resistant *S. aureus*	23 (11.4%)	14 (13.9%)	9 (8.9%)	0.268	1.225 (0.689–2.179)	0.489
Complicated bacteremia	67 (33.2%)	35 (34.7%)	32 (31.7%)	0.654	0.645	1.108 (0.716–1.717)
Infective endocarditis	9 (4.5%)	4 (4.0%)	5 (5.0%)	1.000 ^d^		
Metastatic distant foci	11 (5.4%)	7 (6.9%)	4 (4.0%)	0.352		
Persistent bacteremia	16 (7.9%)	8 (7.9%)	8 (7.9%)	1.000		
Persistent fever > 72 h	9 (4.5%)	5 (5.0%)	4 (4.0%)	1.000		
Vascular prosthesis	7 (3.5%)	3 (3.0%)	4 (4.0%)	1.000 ^d^		
Valve prosthesis	12 (5.9%)	6 (5.9%)	6 (5.9%)	1.000		
Orthopedic prosthesis	12 (5.9%)	5 (5.0%)	7 (6.9%)	0.552		
Intracardiac device	17 (8.4%)	8 (7.9%)	9 (8.9%)	0.800		
60-day overall mortality	28 (13.9%)	6 (5.9%)	22 (21.8%)	0.001		
Treatment failure	31 (15.3%)	9 (8.9%)	22 (21.8%)	0.011	0.660 (0.324–1.345)	0.253

Data are presented as *n* (%) unless otherwise indicated; center effect was included in multivariate analysis. ^a^ *p* values were calculated by x2 except otherwise indicated. ^b^ *p* values and OR were calculated by conditional logistic regression. ^c^ Mann–Whitney U-test. ^d^ Fisher test. ^e^ Osteoarticular, skin and soft tissues, endocarditis or vascular prosthesis, and others (urinary tract, nervous central system).

## Data Availability

No new data were created or analyzed in this study. Data sharing is not applicable to this article.
